# Influence of oxygen on NADH recycling and oxidative stress resistance systems in *Lactobacillus panis* PM1

**DOI:** 10.1186/2191-0855-3-10

**Published:** 2013-01-31

**Authors:** Tae Sun Kang, Darren R Korber, Takuji Tanaka

**Affiliations:** 1 Department of Food and Bioproduct Sciences, College of Agriculture and Bioresources, University of Saskatchewan, 51 Campus Drive, Saskatoon, SK, S7N 5A8, Canada

**Keywords:** *Lactobacillus panis*, NADH oxidase, NADH peroxidase, Electron acceptor, Hydrogen peroxide

## Abstract

*Lactobacillus panis* strain PM1 is an obligatory heterofermentative and aerotolerant microorganism that also produces 1,3-propanediol from glycerol. This study investigated the metabolic responses of *L. panis* PM1 to oxidative stress under aerobic conditions. Growth under aerobic culture triggered an early entrance of *L. panis* PM1 into the stationary phase along with marked changes in end-product profiles. A ten-fold higher concentration of hydrogen peroxide was accumulated during aerobic culture compared to microaerobic culture. This H_2_O_2_ level was sufficient for the complete inhibition of *L. panis* PM1 cell growth, along with a significant reduction in end-products typically found during anaerobic growth. *In silico* analysis revealed that *L. panis* possessed two genes for NADH oxidase and NADH peroxidase, but their expression levels were not significantly affected by the presence of oxygen. Specific activities for these two enzymes were observed in crude extracts from *L. panis* PM1. Enzyme assays demonstrated that the majority of the H_2_O_2_ in the culture media was the product of NADH: H_2_O_2_ oxidase which was constitutively-active under both aerobic and microaerobic conditions; whereas, NADH peroxidase was positively-activated by the presence of oxygen and had a long induction time in contrast to NADH oxidase. These observations indicated that a coupled NADH oxidase - NADH peroxidase system was the main oxidative stress resistance mechanism in *L. panis* PM1, and was regulated by oxygen availability. Under aerobic conditions, NADH is mainly reoxidized by the NADH oxidase - peroxidase system rather than through the production of ethanol (or 1,3-propanediol or succinic acid production if glycerol or citric acid is available). This system helped *L. panis* PM1 directly use oxygen in its energy metabolism by producing extra ATP in contrast to homofermentative lactobacilli.

## Introduction

*Lactobacillus panis* PM1 is an aerotolerant and obligatory heterofermentative microorganism isolated from bioethanol thin stillage, and has been the focus of attention due to its ability to produce 1,3-propanediol (1,3-PDO) during the fermentation of glycerol under anaerobic conditions (Khan et al. 2013). *Lactobacillus panis* belongs to the group III heterofermentative lactobacilli, which includes *L. brevis, L. buchneri* and *L. reuteri,* where the 6-phosphogluconate/phosphoketolase (6-PG/PK) pathway is the primary carbohydrate fermentation pathway (Khan et al. 2013; Luthi-Peng et al. 2002; Pedersen et al. 2004; Veiga-da-Cunha and Foster 1992). In theory, when one glucose molecule is consumed, three NADH and one ATP molecules are generated. Subsequently, one pyruvic acid and one acetyl phosphate molecules accept protons from one and two NADH molecules, respectively, and regenerate NAD^+^. End-products of this metabolism are lactic acid and ethanol, respectively. Overall heterolactic fermentation of glucose through the 6-PG/PK pathway results in 1 mol each of lactic acid, ethanol, and CO_2_ and 1 mol ATP per mol glucose consumed (Kandler 1983).

For heterofermentative lactic acid bacteria (LAB), external electron acceptors can be used as alternate routes to reproduce NAD^+^. The presence or absence of electron acceptors determine whether ethanol (and no more ATP) or acetic acid (and 1 additional ATP) is produced from a glucose molecule (Chen and McFeeters 1986; Condon 1987; McFeeters and Chen 1986; Talarico et al. 1990; Veiga-da-Cunha and Foster 1992). For example, when glycerol exists, the regeneration of NAD^+^ for glucose metabolism can be achieved through the conversion of glycerol to 1,3-PDO using glycerol as the electron receptor (Saxena et al. 2009; Veiga-da-Cunha and Foster 1992). The presence of external electron acceptors, therefore, affects the energy metabolism and end-product profiles, as well as further fermentation applications of LAB.

Molecular oxygen can act as an external electron acceptor and can be advantageous to LAB during cell growth, and its presence in culture conditions greatly influences the physiology of many LAB (An et al. 2010; Condon 1987; Higuchi et al. 2000; Marty-Teysset et al. 2000; Miyoshi et al. 2003). While oxygen itself is not toxic, reactive oxygen species (ROS; including the superoxide anion radical (O_2_^-^), the hydroxyl radical (^·^OH), and hydrogen peroxide (H_2_O_2_)) which are produced during cellular processes can cause a variety of damage to the cell (Condon 1987; Higuchi et al. 2000; Miyoshi et al. 2003). Unlike aerobes and facultative anaerobes, such as *Escherichia coli* and *Salmonella typhimurium,* that have evolved efficient mechanisms for protection against ROS (Farr and Kogoma 1991), LAB lack catalases and functional cytochrome oxidases required for energy-linked oxygen metabolism (An et al. 2011; An et al. 2010; Jansch et al. 2011). Some LAB possess oxidases that utilize molecular oxygen to oxidize substrates such pyruvate or NADH (Condon 1987; Marty-Teysset et al. 2000; Sedewitz et al. 1984). Generally, NADH oxidase is the most common oxidative enzyme in LAB and the systems are often oxygen-inducible (Condon 1987; Higuchi et al. 2000; Komagata 1996; Miyoshi et al. 2003). However, the activity of NADH oxidase can produce hydrogen peroxide (H_2_O_2_) which can then directly oxidize protein cysteinyl residues and inactivate enzymes (Miyoshi et al. 2003). Hydrogen peroxide can also react with cations, such as Fe^2+^ and Cu^2+^, giving rise to hydroxyl radicals via the Fenton reaction (Miyoshi et al. 2003).

Therefore, the presence of oxygen in the growth environment of LAB will induce oxidative stress to which bacteria have various responses mechanisms. A common oxidative stress resistance mechanism found in LAB is a coupled NADH oxidase - NADH peroxidase system (Miyoshi et al. 2003). In these coupled reactions, intracellular oxygen is first used to oxidize NADH into NAD^+^ by NADH oxidase, thereby releasing H_2_O_2_. Subsequently, H_2_O_2_ is reduced to H_2_O by NADH peroxidase (Condon 1987; Higuchi et al. 2000; Miyoshi et al. 2003). However, the activity of NADH peroxidase is generally low (10 to 30 times lower than that of NADH oxidase) in *L. lactis* and has not been detected in some *latobacillus* strains. Thus, cellular H_2_O_2_ detoxification is inefficient in some LAB capable of producing H_2_O_2_ under aerobic conditions (Anders et al. 1970; Komagata 1996).

Our previous research showed that the presence of oxygen during the fermentation of glycerol by *L. panis* PM1 negatively affected cell growth, glucose consumption, and end-product production, including 1,3-PDO. The protection mechanism towards oxidative stress is a key element to optimize *L. panis* PM1 for 1,3-PDO production in biofuel waste material applications. The NADH oxidase - NADH peroxidase system and conversion of glycerol to 1,3-PDO both use NADH as a key factor for their reactions. Therefore, the clarification of the control of oxidative stress by this strain can shed light on how it regulates 1,3-PDO production. In this study, we clearly demonstrated the oxygen-dependent function of NADH oxidase and NADH peroxidase and its involvement in the NAD^+^ regeneration system of *L. panis* PM1.

## Materials and methods

### Chemicals

All chemicals used in this study were ACS grade, or better, and purchased from Sigma-Aldrich (St. Louis, MO, USA).

### Bacterial strains and growth conditions

*Lactobacillus panis* PM1 was isolated from bioethanol thin stillage in our lab (International Depository Authority of Canada; accession number 180310–01). Strain PM1 was cultured at 37°C using commercial MRS medium (BD, Franklin Lakes, NJ, USA) until late log phase and was then transferred to modified MRS medium (mMRS). The mMRS medium consisted of 10 g glucose, 5 g yeast extract, 10 g peptone, 10 g meat extract, 2 g K_2_HPO_4_, 2 g ammonium citrate, 5 g sodium acetate, 100 mg MgSO_4_·7H_2_O, 50 mg MnSO_4_, along with a defined concentration of electron acceptors, such as citric acid (26 mM) or glycerol (160 mM) per litre. The cultures were incubated at 37°C under aerobic or microaerobic conditions. Aerobic and microaerobic cultures were grown using the same medium and temperature. Continuous aeration was provided to aerobic cultures by agitation; whereas, air-tight 15 ml tubes, filled to the two-thirds level, were incubated under static conditions to establish microaerobic conditions. It should be noted that our previous study (Khan et al. 2013) indicated there was little difference in the behaviour of *L. panis* PM1 under anaerobic and microaerobic conditions, thus we did not include anaerobic culture in this study.

### Quantification of H_2_O_2_ production

*Lactobacillus panis* PM1 cells were removed from the culture media using centrifugation (14,000 × *g*, 5 min). Hydrogen peroxide concentrations of the cell-free media were measured in accordance with the Pierce Quantitative Peroxide Assay Kit (Thermo Scientific, Rockford, IL, USA) based on oxidation of ferrous to ferric ion in the presence of xylenol orange.

### RNA preparation

RNA was extracted from *Lactobacillus panis* PM1 cells by the hot phenol extraction method, as described by Oh and So (2003) with minor modifications. Briefly, 10 ml of exponentially-growing bacteria from liquid media were added to a tube containing 1.25 ml of ice-cold ethanol/phenol stop solution (5% water-saturated phenol, pH < 4.5, in 95% ethanol), and harvested by centrifugation for 5 min at 10,000 x g. The cell pellets were resuspended in 600 μl of diethylpyrocarbonate (DEPC)-treated water. Glass beads (0.8 g, 452–600 μm in diameter; Sigma) and 600 μl of pre-warmed acid-hot phenol:chloroform:isoamylacohol (PCI, 25:24:1, v/v) were added to the cell suspensions, and the mixture was incubated at 65°C for 10 min with vigorous vortexing for 30 sec duration every 30 sec. The samples were centrifuged for 10 min at 14,000 x g and then the supernatants (500 μl) were transferred to fresh 1.5-ml micro-tubes containing 500 μl of the pre-warmed PCI and incubated at 65°C for 5 min with vortexing every 30 sec. Samples were then centrifuged for 10 min at 14,000 x g. The aqueous layer (400 μl) was transferred into new 1.5-ml micro-tubes and mixed with 95% ethanol (800 μl) and 3 M sodium acetate (40 μl). The mixtures were kept at −80°C for 30 min and centrifuged at 14,000 x g. The RNA pellet was washed with 70% ethanol and resuspended in 50 μl of RNase-free water. RNA was treated with DNase and purified using the RNeasy kit (Qiagen, Toronto, ON, Canada). The quantity of RNA was determined by measuring the absorbance at 260 nm (optical density (OD) 1 at A_260_ = 40 μg · ml^-1^ RNA), using a DU 800 spectrophotometer (Beckman Coulter, Mississauga, ON, Canada), and its purity was determined by measuring the A_260_/A_280_ ratio.

### Reverse transcription

The primers used in this study were specifically designed by Primer3 (http://frodo.wi.mit.edu/primer3/) for real time RT-PCR applications of *L. panis* PM1 from the nucleotide sequence of the annotation data (Table 1). The RT reaction mixture contained 0.5 μg of total RNA and 0.25 μM of reverse primers of the selected genes. The RT reaction was performed using qScript cDNA SuperMix (Quanta Biosciences, Inc., Gaithersburg, MD, USA) according to the manufacturer’s instructions. The reaction mixture was incubated at 25°C for 5 min and at 42°C for 30 min, and the reaction was terminated by incubation at 85°C for 5 min with a Techne thermal cycler (FTGENE-5D, Techgene, Burlington, NJ, USA).

**Table 1:** Sequences of primers used for qRT_PCR in this study

**Target gene**	**Function**	**Primer**	**Tm (°C)**	**Nucleotide sequence (5’ → 3’)**
*16S rRNA*	16S ribosomal RNA	f16S	58	tggcccaactgatatgac
		r16S	58	ctctcatgcacgttcttctt
*nox*	NADH oxidase	fNOX	60	actggggctgagaagacaga
		rNOX	60	tgctcatcaaaggcagtgac
*npx*	NADH peroxidase	fNPX	60	tcatcaggtgtaacgccaaa
		rNPX	60	taacgcccatcttcaagtcc

### Quantitative PCR

Real-time PCR amplification was performed in a CFX96 real-time detection system (Bio-Rad, Hercules, CA, USA) using SsoFast EvaGreen Supermix (Bio-Rad). The total volume of the PCR master mixture was 20 μl, to which cDNA template equivalent to 25 ng RNA starting material and 0.5 μM of each primer (Table 1) was added. PCR amplification was initiated at 95°C for 30 s followed by 40 cycles of 95°C for 5 s and 60°C for 10 s. Amplification was followed by a melt-curve analysis between 65°C and 95°C using a 0.5°C increment. All sample and primer combinations were assessed in three biological replicates with two technical replicates per biological replicate. A no-template control was used for the negative control PCR, and PCR specificity and product detection were verified by examining the temperature-dependent melting curves of the PCR products and ethidium bromide staining on 1% agarose gel. For relative gene expression, the 2^-ΔΔCt^ method using the 16S rRNA gene as the normalizer was performed as described by Livak and Schmittgen (2001). The steps for calculating the expression ratio are following:

$$\begin{array}{l} {\text{ΔCt}}_{\left(\text{test}\right)}\;=\;{\text{Ct}}_{\left(\text{nox}\;\text{and}\;\text{npx},\;\text{test}\right)}\;\\ \;-\;{\text{Ct}}_{\left(16S\;\text{rNA},\;\text{test}\right)} \end{array}$$

$$\begin{array}{l} {\text{ΔCt}}_{\left(\text{control}\right)}\;=\;{\text{Ct}}_{\left(\text{nox}\;\text{and}\;\text{npx},\;\text{control}\right)}\;\\ \;-\;{\text{Ct}}_{\left(16S\;\text{rNA},\;\text{control}\right)} \end{array}$$

$$\text{ΔΔCt}\;=\;{\text{ΔCt}}_{\left(\text{test}\right)}\;-\;{\text{Ct}}_{\left(\text{control}\right)}$$

Normalized expression ratio of *nox* and *npx*_(test)_ = 2^–ΔΔCt^.

The Real-Time data were processed using CFX Manager Software (Bio-Rad).

### Preparation of crude extracts

*Lactobacillus panis* PM1 cells grown to mid-exponential phase under microaerobic or aerobic conditions were harvested by centrifugation, washed with 100 mM phosphate buffer (pH 7.0), and the cells in pellets were then disrupted using sonication (three times for 1 min with a 3 min rest interval at output level 2, Sonifier 450; Branson, CT, USA) using the same buffer. Crude extract was obtained by centrifugation for 10 min at 14,000 x g, and protein concentration was determined using the Protein Assay Kit (Bio-Rad) with bovine serum albumin (BSA) as a standard.

### Enzyme assay

NADH oxidase and NADH peroxidase activities were determined by measuring the H_2_O_2_ concentration generated and decomposed by the crude extracts, respectively. The assay mixture contained 200 μM of the reduced form of nicotinamide adenine dinucleotide (NADH) and 20 μM flavin adenine dinucleotide (FAD^+^) in 50 mM phosphate buffer at pH 6.0. The assay was carried out at 30°C under aerated or microaerobic conditions. For the NADH peroxidase assay, hydrogen peroxide was added into the above assay mixture to an initial concentration of 30 μM. The concentrations of H_2_O_2_ generated or decomposed were quantified as described above. In these determinations, one unit of activity corresponds to the generation (for NADH oxidase) and decomposition (for NADH peroxidase) of one μmol of H_2_O_2_ in one minute.

### Determination of glucose and end-products

Culture optical density was measured as an index of growth at 600 nm with a DU 800 spectrophotometer. After centrifugation, the supernatant was filtered through 0.22-μm pore size filters and stored at −20°C for HPLC analysis. To quantify the concentration of glucose, organic acids and ethanol, samples were analyzed on an organic acid column (HPX-87H; Bio-Rad) using an HPLC system equipped with a refractive index detector (RID G1362A, 1100 series; Agilent Technologies, Palo Alto, CA, USA). Operating conditions were determined by the method described in the column manual with minor modifications. Filtered culture medium (40 μl) was loaded on the column and eluted with 5 mM sulfuric acid at a flow rate of 0.6 ml/min at 55°C for 30 min.

### Statistical analysis

For growth experiments and determinations of H_2_O_2_ concentrations, data are presented as the mean values calculated from at least three independent experiments. For activities of NADH oxidase and NADH peroxidase, standard errors of the means from at least three independent experiments were also calculated and presented. Differences in culture and enzyme assay conditions with NADH oxidase or NADH peroxidase activity (unit/mg protein) were analyzed by the *t* test (Mann–Whitney test) for two groups or the one-way ANOVA test (Kruskal-Wallis test) for three groups using GraphPad Prism 5.0 software (GraphPad Software, Inc., San Diego, CA, USA). *P* < 0.05 was considered significant.

## Results

### Influence of oxygen on the physiology of *L. panis* PM1

**Figure 1:**
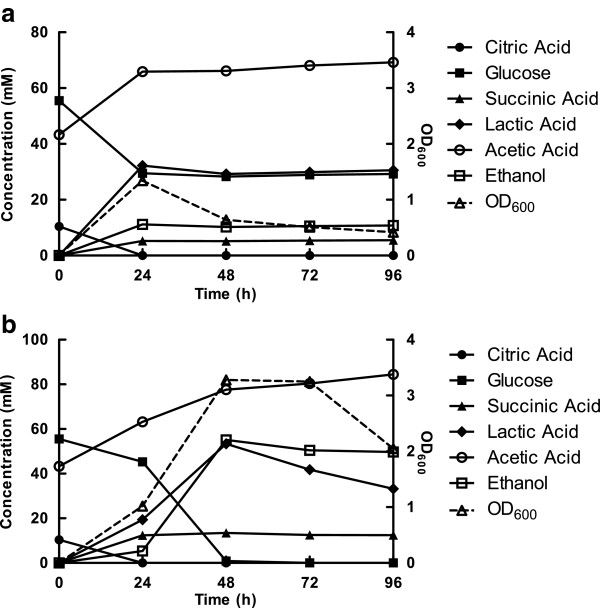
**Effect of oxygen and time on growth and end-product formation in *L. panis *PM1.** Growth response (OD_600_) and end-product formation of *L. panis* PM1 cultivated in mMRS under aerobic (**a**) and microaerobic (**b**) conditions. Samples for cell density and HPLC analysis were withdrawn from the cultures after 24, 48, 72 and 96 hours.

The rates of growth during the first 24 hours of culture were similar between aerobic and microaerobic *L. panis* PM1; however, the aerobically cultured *L. panis* PM1 entered stationary phase earlier than the microaerobic culture (Figures 1a and b). This early entry into stationary phase was associated with a halt in production of end-products, but not with glucose depletion (as approximately 30 mM glucose remained after 24 hours) (Figure 1a), indicating that glucose concentration was not a critical cause of the cellular growth interruption. Unlike aerobically cultured samples, the microaerobic cultures consumed all available glucose (55 mM) within 48 hours and produced nearly-equimolar amounts of lactic acid and ethanol (Figure 1b), revealing a typical heterolactic fermentation of glucose through the 6-PG/PK pathway. Also, the cells grown under microaerobic conditions were observed to consume lactic acid during stationary phase, reducing the concentration of lactate from 53 mM to 33 mM. In contrast, aerobic cultures did not utilize lactic acid after cessation of glucose consumption (Figure 1a). Furthermore, the ratio of ethanol production to glucose consumption (11:26 mM) was less during aerobic culture than during microaerobic culture (55:55 mM). These results indicated that, under aerobic conditions, an alternate metabolic route re-oxidized NADH through the 6-PG/PK pathway while not forming ethanol.

### Production of H_2_O_2_ by aerobic culture and its effects on bacterial growth

**Figure 2:**
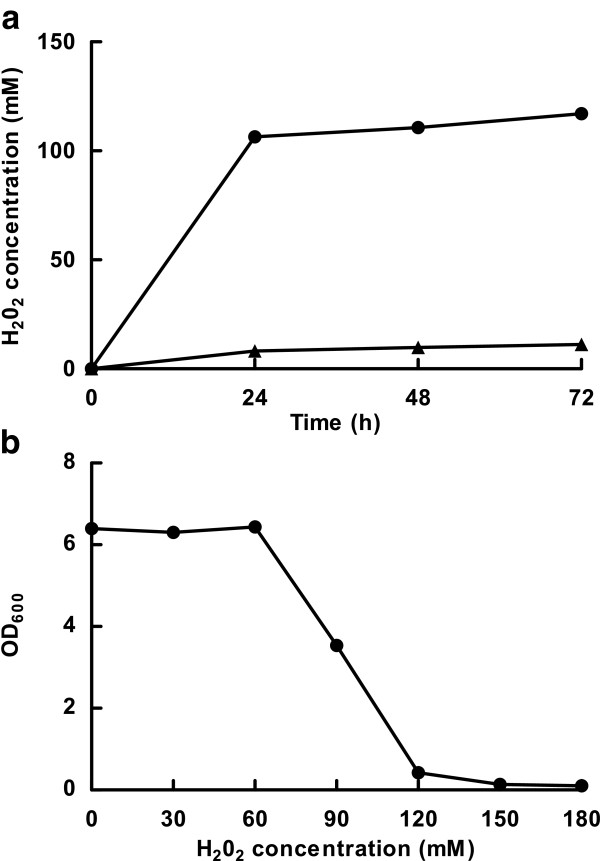
**Effect of oxygen and time on the accumulation of hydrogen peroxide and growth by *L. panis*.** Growth response (OD_600_) and H_2_O_2_ production by *L. panis* PM1 cultured in mMRS under aerobic (circles) and microaerobic (triangles) conditions after 24, 48, and 72 hours (**a**). The inhibitory concentrations of H_2_O_2_ were determined from *L. panis* PM1 cultures grown in MRS broth containing H_2_O_2_ at concentration of 0, 30, 60, 120, 240, and 480 μM for 2 days under microaerobic conditions. Optical density was measured at 600 nm with a spectrophotometer (**b**).

Aerobic culture resulted in the production of ten-fold higher concentrations of H_2_O_2_ than during microaerobic culture. Rapid accumulation of H_2_O_2_, reaching approximate 100 μM, was achieved in the first 24 hours of aerobic culture (Figure 2a). The concentration of H_2_O_2_ necessary to completely inhibit the growth of *L. panis* PM1 was determined to be approximately 120 μM H_2_O_2_ (Figure 2b). Accumulation of H_2_O_2_ reached close to this inhibitory concentration level within 24 hours of aerobic culture. Concurrently, a reduction of cell density was observed after 24 hours aerobic culture (Figure 1b). This data, therefore, indicated a clear association between H_2_O_2_ produced under aerobic conditions and the early entrance of *L. panis* PM1 into stationary phase.

### Specific activities of NADH oxidase and NADH peroxidase

The whole genome data of *L. panis* PM1 (unpublished draft data) revealed only candidate genes for NADH oxidase and NADH peroxidase; whereas, other protective enzyme genes that might respond to the toxic effects caused by oxygen were not detected. The expression levels of these two genes were compared under aerobic and microaerobic conditions by qRT-PCR. It was determined that *nox* (NADH oxidase gene) and *npx* (NADH peroxidase gene) were expressed at similar levels under both culture conditions (Table 2) even though *L. panis* PM1 was shown to produce lethal levels of hydrogen peroxide under aerobic, but not microaerobic, conditions. The levels of activity of NADH oxidase and NADH peroxidase were measured from the cells grown under microaerobic and aerobic culture conditions. The specific activity of NADH oxidase was comparable (*P* > 0.1) under both aerobic and microaerobic cultures (Table 2). Interestingly, the activities of NADH oxidase were dependent on availability of oxygen in the respective assay reactions. When the specific activities of NADH oxidase were compared between aerated and non-aerated assay conditions, significant differences were observed (*P* < 0.05). Higher activities of NADH oxidase were observed in aerated assay than non-aerated assay for both aerobically- and microaerobically-grown cultures (158.8 ± 7.6 vs. 92.5 ± 2.2 and 144.0 ± 2.0 vs. 103.1 ± 5.6 units/mg, respectively). In contrast to NADH oxidase, NADH peroxidase activity was only detected in aerobic cultures. Enzyme assay conditions significantly (*P* < 0.05) affected the levels of activity of NADH peroxidase in the opposite direction of NADH oxidase; higher enzyme activity was observed under non-aerated assay conditions (148.3 ± 9.7 vs. 197.3 ± 1.7 units/mg) (Table 2).

**Table 2:** **The specific activities of NADH oxidase and NADH peroxidase of *L. panis *PM1**

**Enzyme sources **^**a**^	**Enzyme assay conditions**	**Enzyme activity **^**b**^		**Relative gene expression level **^**j**^	
		**NADH oxidase**	**NADH peroxidase**	***nox***	***npx***
Aerobic culture	Aerated condition	158.8 ± 7.6 ^d^	148.3 ± 9.7 ^h^	1.50 ± 0.30	1.16 ± 0.28
Microaerobic culture		144.0 ± 2.0 ^e^	N.D ^c^	1.00 ± 0.22	1.00 ± 0.10
Aerobic culture	Non-aerated condition	92.5 ± 2.2 ^f^	197.3 ± 1.7 ^i^		
Microaerobic culture		103.1 ± 5.6 ^g^	N.D ^c^		

^*a*^*. Lactobacillus panis* PM1 was cultured in mMRS for 24 hours under aerobic or microaerobic conditions for enzyme assays. ^b^. Values represent specific activities (unit/mg protein). One unit is defined as one micromole of H_2_O_2_ generated (for NADH oxidase) or decomposed (for NADH peroxidase) per minute. ^c^. Minus values where H_2_O_2_ generating ability was higher than H_2_O_2_ decomposing ability are defined as non-detectable (N.D) in NADH peroxidase assay. ^d^. Significantly different to f, *P* < 0.05. ^e^. Significantly different to g, *P* < 0.05. ^h^. Significantly different to i, *P* < 0.05. ^j^. Microaerobic culture was used for control sample for relative gene expression by qRT-PCR.

### Role of oxygen in oxidative stress

**Figure 3:**
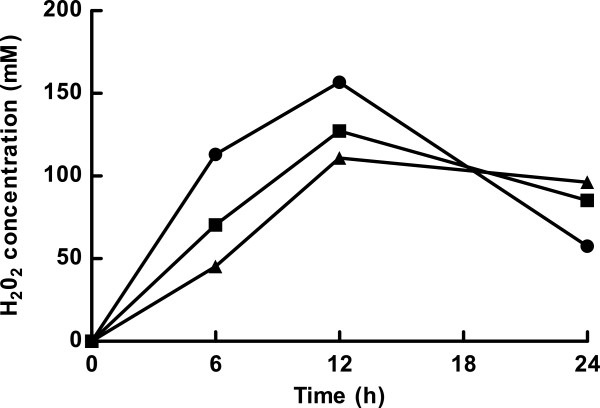
**Effect of oxygen availability on accumulation of hydrogen peroxide in *L. panis *PM1 culture media.** H_2_O_2_ production by *L. panis* PM1 cultured in mMRS in 15 ml conical tubes with 3 (circles), 6 (squares), and 9 (triangles) ml mMRS, respectively, under aerobic conditions. H_2_O_2_ concentration was measured at 6, 12, and 24 hours.

Oxygen availability in the culture media directly affected the coupled NADH oxidase - NADH peroxidase system of *L. panis* PM1, controlling the accumulation of H_2_O_2_ under aerobic conditions. When *L. panis* PM1 was cultured in 15-ml conical tubes containing 9, 6, and 3 ml mMRS under aerobic conditions (in order to incrementally-increase oxygen availability in the aerobic cultures), the H_2_O_2_ accumulation was greatest and most rapid in the 3-ml culture, reaching a maximal value by 12 hours in all samples (Figure 3). The H_2_O_2_ concentration decreased after 12 hours in all samples; however, the degree of H_2_O_2_ decomposition occurred in proportion to oxygen availability in the culture media (63% in the 3-ml culture, 33% in the 6-ml culture, and 13% in the 9-ml culture). The final amount of cell growth was in accordance with the amount of H_2_O_2_ accumulated in the culture media until 12 hours (Table 3 and Figure 3). NADH oxidase activity was constitutively-expressed during the early stages of cell culture, and high NADH oxidase activities were determined in the all 6-hour aerobic cultures (approximately 100 units/mg). The activity of this enzyme increased in a time-dependent manner until after 24 hours of culture. The specific activities of NADH oxidase did not show significant differences in the three aerobic culture conditions (*P* > 0.1). In contrast to NADH oxidase, NADH peroxidase specific activity was only observed in the 3-ml 24-hour culture (Table 3). This data clearly demonstrated that NADH peroxidase activity was induced according to oxygen availability that also elevated the production of H_2_O_2_ by NADH oxidase.

**Table 3:** The result of cell growth and specific activities of NADH oxidase and NADH peroxidase according to oxygen availability

**Culture Vol.**	**3 ml**	**6 ml**	**9 ml**
Cell Growth (OD_600_) ^a^	0.59	1.18	2.26
NADH oxidase activity ^b^	177.6 ± 3.0	193.2 ± 2.5	192.8 ± 5.7
NADH peroxidase activity ^b^	120.1 ± 12.51	N.D	N.D

^*a*^*. Lactobacillus panis* PM1 was cultured in mMRS under aerobic conditions, and samples for cell growth and enzyme assays withdrawn from the cultures after 24 hours. ^b^. Values represent specific activities (unit/mg protein). One unit is defined as one micromole of H_2_O_2_ generated (for NADH oxidase) or decomposed (for NADH peroxidase) per minute. NADH oxidase and NADH peroxidase assays were carried out under aerated and non-aerated conditions, respectively.

### The change of NADH flux by NADH oxidase

**Figure 4:**
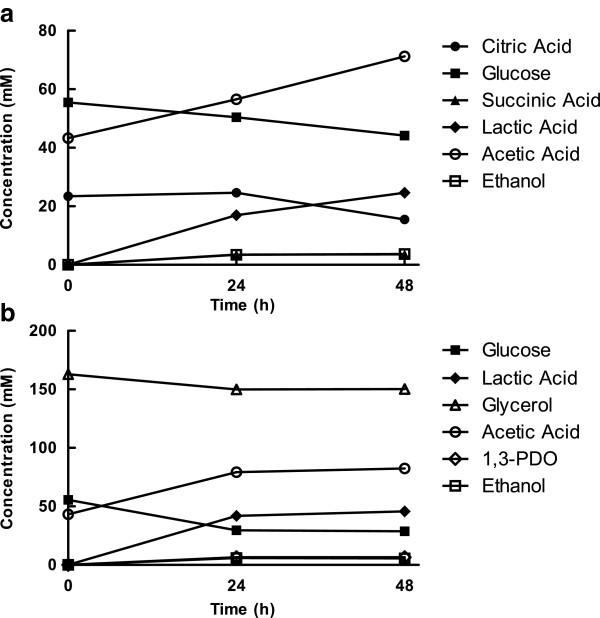
**Effect of external electron acceptors on end-product formation by *L. panis *PM1.** End-product formation by *L. panis* PM1 cultured in mMRS containing either 24 mM citric acid (**a**) or 160 mM glycerol (**b**) as external electron acceptor under aerobic conditions. Samples for HPLC analysis were withdrawn from the cultures after 24 and 48 hours.

Oxygen was a preferred electron acceptor for glycerol or citric acid and changed the flux of NADH for reoxidation in *L. panis* PM1. Inhibitory levels of H_2_O_2_ were accumulated following 24 hour culture of *L. panis* PM1 in mMRS containing either citric acid (24 mM) or glycerol (160 mM) as electron acceptors under aerobic conditions (124 and 120 μM H_2_O_2_, respectively). The consumption of glucose (11 and 27 mM in citric acid and glycerol media, respectively) and production of ethanol (4 and 6 mM in citric acid and glycerol media, respectively) were suppressed similar to that observed in aerobic cultures lacking additional electron accepters (Figure 4). In addition, little citric acid (7 mM) or glycerol (13 mM) was consumed. The amount of lactic acid produced correlated only with the amount of glucose utilized. Considering the amount of citric acid or glycerol consumed and acetic acid produced (28 and 39 mM, respectively) in the culture media, it appeared that citric acid and glycerol contributed only slightly to an increase in acetic acid production and utilization for NADH recycling.

## Discussion

We previously reported the aerotolerant nature of *L. panis* PM1 and its ability to use glycerol as the means of NADH recycling in the absence of oxygen (Khan et al. 2013). However, the presence of oxygen prevented 1,3-PDO formation and thus markedly-affected NADH recycling in this strain. In this study, the influence of oxygen on NADH recycling system and the oxidative stress resistance mechanism in its aerotolerance was investigated. Moreover, the metabolic profile was further investigated to understand how oxidative stress resistance mechanisms of *L. panis* PM1 influenced the profile of metabolic end-products.

During aerobic culture, *L. panis* PM1 prematurely entered into a stationary phase without depleting glucose (Figure 1a). This early entry into stationary phase was also associated with a ten-fold higher accumulation of H_2_O_2_ compared with microaerobic culture (Figure 2a). Therefore, the accumulation of H_2_O_2_ in aerobic culture was an apparent reason for the early cessation of growth. Anaerobic metabolism theoretically makes one ethanol per every glucose consumed, but the presence of oxygen altered this pattern to less than 1:1 ratio. These observations suggested that H_2_O_2_ could be a main end-product of an alternate pathway for NADH recycling under aerobic conditions, and that this could compete with NAD^+^-regeneration through ethanol production.

The production of H_2_O_2_ by LAB grown under aerobic conditions is commonly the result of flavoprotein oxidases, including NADH oxidase, pyruvate oxidase, α-glycerophosphate oxidase, and superoxide dismutase (Condon 1987). However, candidate genes for these enzymes were not found in the draft genome data of *L. panis* PM1, with the exception of NADH oxidase. Pyruvate oxidase has been documented in a few species of lactobacilli and is known to convert pyruvate to CO_2_ and acetyl phosphate, along with the formation H_2_O_2_ (Condon 1987). Pyruvate oxidase has its highest activity during the early stationary phase of growth and is induced and repressed by oxygen and glucose, respectively, in *L. plantarum* (Saxena et al. 2009; Veiga-da-Cunha and Foster 1992). However, the presence of pyruvate oxidase does not adequately explain the early entry into stationary phase observed during the aerobic culture of *L. panis* PM1. Our results showed that most of pyruvate produced during glucose consumption was used to produce lactate in aerobic culture (Figure 1a), indicating that pyruvate oxidase apparently removed little pyruvate from this pathway. NADH oxidase is the most common enzyme responsible for the production of H_2_O_2_ from oxygen and is highly-active in LAB (Condon 1987; Higuchi et al. 2000; Tachon et al. 2011). LAB are known to possess either a NADH: H_2_O_2_ or a NADH: H_2_O oxidase, or sometimes both (Condon 1987; Higuchi et al. 2000). Final products of the reaction of NADH oxidase include either NAD^+^ and H_2_O_2,_ or NAD^+^ and H_2_O, depending on whether two- or four-electrons are transferred by NADH: H_2_O_2_ oxidase or NADH: H_2_O oxidase (Condon 1987; Higuchi et al. 2000; Miyoshi et al. 2003). Our results showed that the crude extract from *L. panis* PM1 cultured under aerobic and microaerobic conditions could directly produce H_2_O_2_ using oxygen as a substrate, and the activity of the enzyme was found to increase with the addition of FAD^+^ as well as aeration of the assay mixture (approximately 1.5 fold). These results indicated that the NADH oxidase in *L. panis* PM1 was a NADH: H_2_O_2_ oxidase and a flavoprotein-like NADH oxidase, as seen in other gram-positive bacteria (Komagata 1996; Marty-Teysset et al. 2000; Tachon et al. 2011).

Most LAB can respond (and protect themselves) to high concentrations of H_2_O_2_ produced through their oxidase enzymes during sugar fermentation (Higuchi et al. 2000). In fact, most LAB possess NADH peroxidase or pseudocatalase, and superoxide dismutase exists in some LAB (Condon 1987). These enzymes can enable LAB to overcome otherwise-lethal concentrations of hydrogen peroxide. The annotation data of the *L. panis* PM1 genome sequence and the results of the enzyme assays of NADH oxidase and NADH peroxidase suggest that these enzymes are main factors in oxidative stress resistance. The levels of accumulated H_2_O_2_ in the culture media could be accounted for by the differences in the activities of NADH peoxidase and NADH oxidase. Our qRT-PCR analyses showed that oxygen did not regulate *nox* and *npx* at the transcriptional-level, and mainly affected enzyme activities in *L. panis* PM1 (Table 2). While transcription levels were similar, activity assays exhibited that NADH peroxidase was positively-activated by oxygen but required a long induction time to express activity contrary to NADH oxidase. The oxygen-availability analyses indicated that higher oxygen availability in the 3-ml culture could provide higher amounts of substrate (oxygen) for NADH oxidase, resulting in greater accumulation of H_2_O_2_ in the first 12 hours. In the subsequent 12 hours, the accumulated H_2_O_2_ was decomposed by NADH peroxidase activity. The degree of degradation of H_2_O_2_ was dependent on NADH peroxidase activity, and the amount of activity was in proportion with oxygen availability (Figure 3 and Table 3). Therefore, we concluded that a coupled NADH oxidase - NADH peroxidase system, regulated by oxygen availability, was a key oxidative stress resistance mechanism in *L. panis* PM1.

Accumulation of H_2_O_2_ by NADH oxidase has been reported in group I homofermentative lactobacilli, like *L. delbrueckii,* where approximate 97% of NADH was reoxidized by lactate dehydrogenase and NADH oxidase accounted for only 3% of NADH reoxidation (Marty-Teysset et al. 2000). Thus, NADH recycling in group I LAB depends on a pyruvate supply from glycolysis, rather than oxygen. Unlike homofermentative lactobacilli, the presence of electron acceptors, such as oxygen, citric acid, or glycerol, directly influenced the flux of NADH reoxidation in *L. panis* PM1. In our other studies, when *L. panis* PM1 was cultured in mMRS containing citric acid (24 mM) and glycerol (150 mM) under microaerobic conditions, the major changes in end-product formation included a decrease in ethanol, an increase in acetic acid, and the production of succinic acid (19 mM) and 1,3-PDO (88 mM), respectively (unpublished data). The results of HPLC analyses in the present study showed that aerobic conditions negatively-affected the production of ethanol relative to glucose consumption, regardless of the presence of electron acceptors (Figures 1a and 4). Also, when *L. panis* PM1 was cultured under aerobic conditions in mMRS containing citric acid and glycerol, oxygen was used as the preferred electron acceptor, resulting in a shift of NADH flux along with a significant decrease of the production of succinic acid (4 mM) and 1,3-PDO (7 mM) (Figure 4). This data indicated that the activity of NADH oxidase was a key mechanism for the reoxidation of NADH during growth in aerobic culture.

In addition to oxidative stress responses, NADH oxidase can also help *L. panis* PM1 use oxygen during energy metabolism, directly. That is, the shift of NADH recycling with molecular oxygen redirected acetyl phosphate, which normally would be used to produce ethanol, to the formation of acetic acid. This acetic acid production via acetate kinase can stoichiometrically generate ATP (Condon 1987). Thus, O_2_-directed NADH recycling should be advantageous with respect to energy metabolism. However, regeneration of NAD^+^ via NADH oxidase in *L. panis* PM1 led to overproduction of H_2_O_2_ with subsequent negative effects on growth and end-product formation. Our findings indicate that varied oxygen availabilities of culture environments would greatly affect energy metabolism as well as oxidative stress of *L. panis* PM1. The formation of 1,3-PDO is a main route for NADH reoxidation in the presence of glycerol under anaerobic conditions; whereas, under aerobic conditions, NADH recycling largely occurs through NADH oxidase activity. The present study indicates that energy metabolism via the NADH oxidase system explains why *L. panis* PM1 fails to produce 1,3-PDO under aerobic conditions.

## Competing interest

The authors declare that they have no competing interest.
